# Targeted Therapies for Parkinson's Disease: From Genetics to the Clinic

**DOI:** 10.1002/mds.27414

**Published:** 2018-04-27

**Authors:** S. Pablo Sardi, Jesse M. Cedarbaum, Patrik Brundin

**Affiliations:** ^1^ Neuroscience Therapeutic Area Sanofi Framingham MA USA; ^2^ Neurology Early Clinical Development Biogen Cambridge MA USA; ^3^ Center for Neurodegenerative Science Van Andel Research Institute Grand Rapids MI USA

**Keywords:** *SNCA*, *GBA LRRK2*, α‐synuclein, glucocerebrosidase, clinical trial

## Abstract

The greatest unmet medical need in Parkinson's disease (PD) is treatments that slow the relentless progression of symptoms. The discovery of genetic variants causing and/or increasing the risk for PD has provided the field with a new arsenal of potential therapies ready to be tested in clinical trials. We highlight 3 of the genetic discoveries (α‐synuclein, glucocerebrosidase, and leucine‐rich repeat kinase) that have prompted new therapeutic approaches now entering the clinical stages. We are at an exciting juncture in the journey to developing disease‐modifying treatments based on knowledge of PD genetics and pathology. This review focuses on therapeutic paradigms that are under clinical development and highlights a wide range of key outstanding questions in PD. © 2018 The Authors. Movement Disorders published by Wiley Periodicals, Inc. on behalf of International Parkinson and Movement Disorder Society.

Parkinson's disease (PD; online mendelian inheritance in man (OMIM) https://www.omim.org/entry/168600) is the most common neurodegenerative movement disorder, affecting 1 to 2 per 1000 of the population at any time. The current medical management of PD aims at controlling signs and symptoms for as long as possible while minimizing adverse effects. Therapies directed at controlling the relentless progression of the disease are desperately needed.^1^, ^2^

The development of therapeutics for PD is at the cusp of undergoing a revolution. Several recent developments have led to human studies that test novel therapeutic targets with the hope of slowing progression of symptoms. This revolution comes in the wake of several genetic discoveries, decades of academic and pharmaceutical research, patient volunteerism in natural history and biomarker studies, and the development of novel biosensor technologies. These advances provide a springboard to evaluate a new class of therapies aimed at slowing PD progression. This review summarizes the therapeutic paradigms under clinical development that originated from genetic discoveries in the past 2 decades. We also highlight the key outstanding questions that are being addressed to advance this new class of therapies.

The etiology of idiopathic PD is multifactorial, likely arising from a combination of polygenic inheritance, environmental exposures, and gene–environment interactions. Although monogenic, inherited forms of PD are rare and constitute around 5% to 10% of all cases, 20% of patients with PD report having 1 affected first‐ or second‐degree relative.^3^ The initial discovery of α‐synuclein gene mutations and multiplications as a cause of PD more than 20 years ago have been followed by intensive research and the identification of numerous genes linked to PD pathogenesis.^3^ This review summarizes how these initial genetic findings enabled targeting therapies for α‐synuclein (*SNCA*), glucocerebrosidase (*GBA*), and leucine‐rich repeat kinase (*LRRK2*), as these are the most advanced in clinical development (Table 1).

**Table 1:** Genetic‐based targeted therapies currently being tested in PD patients^a^

Gene	Targeting mechanism	Drug	Therapeutic modality	Mechanism of action	Target population (n)	Status	ClinicalTrials.gov ID	Sponsor
*SNCA*	Decrease α‐synuclein aggregation	NPT200‐11	Small molecule	Inhibition of α‐synuclein misfolding	HV (55)	Phase I	NCT02606682	Neuropore Therapies and UCB Pharma
		NPT088	Biologic	Reduction of α‐synuclein aggregation	AD (66)^b^	Phase I	NCT03008161	Proclara
	Increase α‐synuclein degradation	Nilotinib	Small molecule	Inhibition of c‐Abl	Mild PD(75)	Phase II	NCT02954978	Georgetown University
					Early and mild PD (135)	Phase II	NCT03205488	Northwestern University, MJFF, Cure Parkinson's Trust and Van Andel Institute
	Decrease extracellular α‐synuclein	RO7046015	Biologic	Passive immunization	Early PD (300)	Phase II	NCT03100149	Prothena and Roche
		BIIB054	Biologic	Passive immunization	Early PD (311)	Phase II	NCT03318523	Biogen
		MEDI1341	Biologic	Passive immunization	HV (40)	Phase I	NCT03272165	AstraZeneca and Takeda
		PD01A, PD03A	Biologic	Active immunization	Early PD (36)	Phase I	NCT02267434	AFFITOPE
*GBA*	GCase activation	Ambroxol	Small molecule	GCase activation	GBA‐PD (10) PD (10)	Phase II	NCT02941822	UCL and Cure Parkinson's Trust
					PDD (75)	Phase II	NCT02914366	Lawson Health Research Institute and Weston Foundation
	Reduction of *GBA*‐related GSLs	Venglustat	Small molecule	Glucosylceramide synthase inhibitor	GBA‐PD (243)	Phase II	NCT02906020	Sanofi
*LRRK2*	LRRK2 kinase inhibition	DNL201	Small molecule	Kinase inhibitor	N/A	Phase I	N/A	Denali

*SNCA*, α‐synuclein; *GBA*, glucocerebrosidase; *LRRK2*, leucine‐rich repeat kinase; HV, healthy volunteer; AD, Alzheimer's disease; PDD, PD dementia; GBA‐PD, glucocerebrosidase‐PD; GCase, glucocerebrosidase; GSLs, glycosphingolipids; UCB, Union Chimique Belge; MJFF, The Michael J. Fox Foundation; UCL, University College London; N/A, Not available; c‐Abl, (ABL1, Abelson tyrosine kinase).

a From ClinicalTrials.gov, accessed February 2018.

b Phase I in Alzheimer's disease patients might support advancement into Phase 2/3 for PD.

## Therapeutic Approaches Targeting α‐Synuclein

**Figure 1:**
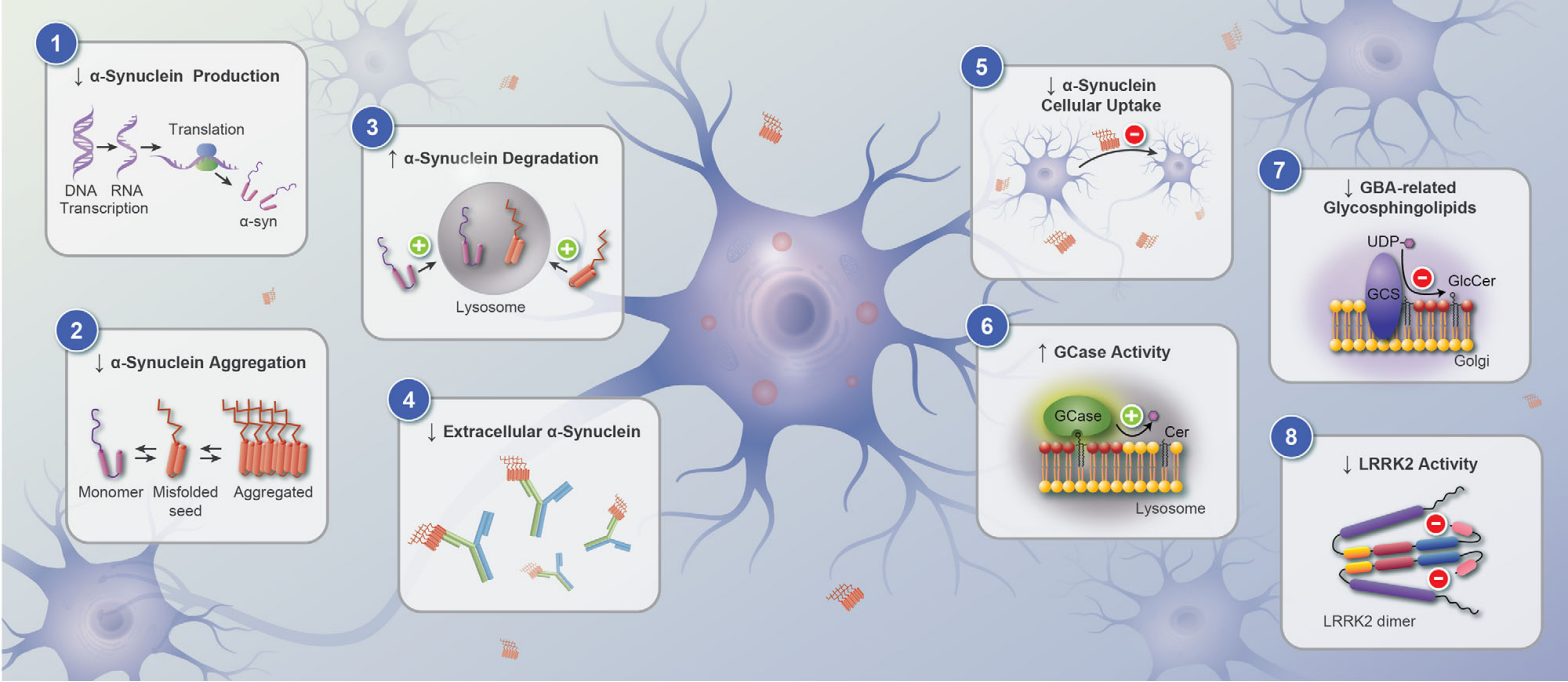
Potential mechanisms for genetic‐based investigational therapies for Parkinson's disease targeting α‐synuclein (*SNCA*), glucocerebrosidase (*GBA*), and leucine‐rich repeat kinase (*LRRK2*). Targeting *SNCA*: (1) reducing α‐synuclein production, (2) decreasing α‐synuclein aggregation, (3) enhancing autophagy, (4) reducing availability of extracellular aggregates, (5) inhibiting cellular uptake of α‐synuclein. Targeting the *GBA* pathway: (6) increasing glucocerebrosidase (GCase) activity, (7) modulating *GBA*‐related glycosphingolipids. Targeting *LRRK2*: (8) LRRK2 kinase inhibitors.

Multiple lines of evidence support a pivotal role of α‐synuclein in PD pathogenesis.^4^ The presence of aggregated α‐synuclein in specific brain regions is the hallmark of PD and suggests a central role for this protein in the sporadic disorder. In neurons, α‐synuclein normally participates in the regulation of neurotransmitter release from the presynaptic terminals.^5^ Mutations and multiplications in *SNCA* cause familial PD.^3^ Recently, genomewide association studies have consistently identified common genetic variants close to the *SNCA* locus that increase the risk for idiopathic PD.^6^, ^7^ A large body of evidence suggests that the accumulation of α‐synuclein plays a key role in PD. The formation of α‐synuclein oligomers and fibrils that are ineffectively cleared by lysosomal or the ubiquitin proteasome systems result in aggregation and Lewy body pathology.^8^ Consequently, a wide range of approaches has been developed in attempts to prevent α‐synuclein aggregation and cell‐to‐cell propagation of misfolded α‐synuclein species with the ultimate aim of slowing PD progression (Fig. 1). In this section, we briefly review some of these approaches and focus on those where there is a clear path to clinical application or where clinical trials have even been initiated.

### Challenges Facing α‐Synuclein Therapeutic Approaches

Before describing the different approaches, it is pertinent to point out that the challenges with therapies aimed at preventing α‐synuclein aggregation and propagation of α‐synuclein aggregates are numerous.

First, there is no naturally occurring animal model that naturally replicates α‐synucleinopathy in humans. Therefore preclinical testing of therapies targeting α‐synuclein is confined to models that are at best correlative to the human condition. The currently most used mammalian models of α‐synucleinopathy fall into 3 main categories. Several transgenic mouse models that overexpress either wild‐type or mutant α‐synuclein have been found to exhibit α‐synuclein aggregates, but the anatomical patterns (which are linked to the promoter used in the transgene) typically do not mimic that seen in PD, and nigral neurodegeneration is not a common feature.^9^ Another approach is viral vector‐mediated overexpression of α‐synuclein targeted to the nigrostriatal pathway, which leads to some degeneration of nigral dopamine neurons but is labor intensive and has a relatively high variability.^10^ A more recently developed type of model involves the injection of α‐synuclein fibrils into the brain, which triggers a protracted development of α‐synuclein aggregates in interconnected brain regions, followed by neurodegeneration in some of the affected brain regions (eg, loss of nigral dopamine neurons after injection of fibrils into the striatum).^11^, ^12^ An advantage with this last paradigm is that it is possible to mimic some of the neuropathology and functional deficits seen in prodromal PD by injecting the fibrils into the olfactory bulb.^13^, ^14^

A second major challenge is that there currently is no established method to assess target engagement in the brain for therapies that target α‐synuclein.^15^, ^16^, ^17^ The key species of α‐synuclein aggregates that is pathogenic and causes neurodegeneration remains to be established, and consequently, it is unknown if a therapy needs to reduce the formation of oligomers or fibrils or even if reducing availability of monomeric building blocks of the aggregates is sufficient.

Third, it is currently not possible to image the severity of α‐synuclein pathology in patient brains, and there is no biofluid‐based biomarker that can assess the level of α‐synuclein pathology in patient brains^18^ Without reliable in vivo markers of α‐synuclein pathology in humans, clinical trials with novel therapies targeting α‐synuclein pathology have to rely only on correlative clinical endpoints, and as a consequence it is difficult to, for example, define optimal dosing. Availability of an α‐synuclein imaging agent would represent a great advantage, allowing the selection of study subjects with known α‐synuclein pathology and monitoring of treatment‐related changes, similarly to a recently reported trial of Aducanumab for early Alzheimer's disease.^19^

Fourth, it is not known when during the natural history of PD the first intracerebral α‐synuclein pathology appears and whether there is a “point of no return” beyond which the damage to the neural systems affected by synucleinopathy can no longer be protected or revived, even with the most effective therapies. As mentioned earlier, it is currently believed that α‐synuclein pathology begins to develop during the PD prodrome,^20^ and by the time motor symptoms have appeared the aggregates are widespread in the brain.

Notwithstanding these challenges, research into therapies targeting α‐synuclein is vibrant and is making significant progress on numerous fronts. The approaches to stop α‐synuclein aggregation can be roughly divided into 5 categories based on the primary mechanism of action (Fig 1). There are methods to (i) reduce α‐synuclein production, (ii) reduce intracellular α‐synuclein aggregation, (iii) increase intracellular α‐synuclein degradation, (iv) promote degradation of extracellular α‐synuclein, and (v) inhibit neuronal uptake of extracellular α‐synuclein.

### Reducing α‐Synuclein Production

The observation that α‐synuclein gene multiplications lead to α‐synucleinopathy and neurological disease in humans is the strong premise for creating therapies that reduce α‐synuclein expression. It has been suggested that minor increases in neuronal α‐synuclein levels might explain why certain polymorphisms adjacent to the α‐synuclein locus are associated with increased PD risk, which adds further support to the notion that reducing α‐synuclein levels could be beneficial. This reduction of total protein amounts can be achieved by gene silencing mechanisms targeting α‐synuclein mRNA levels (Fig. 1). A recent report by IONIS Pharmaceuticals and Biogen on the success in a phase III trial using antisense oligonucleotide therapy in spinal muscular atrophy^21^ and news of safety and target engagement with antisense therapy in a phase I trial in Huntington's disease has fueled optimism for antisense therapies in additional neurological conditions.^22^, ^23^ Several approaches to reduce α‐synuclein expression using viral vector‐mediated production of small interfering RNA against α‐synuclein in the substantia nigra have been successfully tested in animal models.^24^ Antisense oligonucleotides designed to promote Ribonuclease H‐mediated degradation of α‐synuclein messenger RNA have been shown to safely lower levels of α‐synuclein message and protein and to protect nigral dopaminergic neurons in rodent experiments.^25^ One potential caveat is that in some of the animal model paradigms, a marked downregulation of α‐synuclein was associated with nigral degeneration, that is, the opposite of the predicted effect.^26^ Current studies are trying to clarify if this toxicity is related to the degree of α‐synuclein knockdown or specific properties of the RNA lowering mechanism and whether the system can be fine‐tuned so that the targeted neurons maintain levels of α‐synuclein needed for survival.

A recent intriguing observation comes from a study suggesting that β2‐adrenergic agonists (eg, clenbuterol and salbutamol) can reduce α‐synuclein levels, purportedly by altering histone acetylation at the α‐synuclein gene promoter and enhancer regions and that this is neuroprotective in cell and animal models of PD.^27^ Further evidence for the relevance of these findings was provided by the analysis of a large Norwegian epidemiological cohort demonstrating that the use of salbutamol against asthma is coupled with the reduced lifetime risk of subsequently developing PD. Remarkably, the use of a β2 antagonist (propranolol) actually increased PD risk, supporting the underlying biology.^27^ However, the interpretation of these results must take into account the inverse relationship between smoking and PD as well as the fact that propranolol is often prescribed for treatment of essential tremor,^28^ a known risk factor for PD. These findings warrant further studies on the effects of β2‐adrenergic agonists in other population cohorts and in animal models of α‐synucleinopathy.

### Aggregation Inhibitors

Intrabodies are small fragments of antibodies (140‐250 amino acids in size) that can enter cells. They can be engineered to bind monomeric α‐synuclein and inhibit oligomerization. Such intrabodies have been shown to reduce α‐synuclein aggregates and nigral neurodegeneration in rodents with viral vector‐mediated α‐synuclein overexpression.^29^ Two other programs driven by industry also aim to interfere with α‐synuclein aggregation. Neuropore Therapies has partnered with UCB Pharma to develop the small molecule NPT200‐11, which is reported to interfere with α‐synuclein interaction with membranes and retard oligomerization, which has been shown to inhibit development of α‐synuclein pathology in a mouse model. A single‐ascending dose study was completed in early 2016 (ClinicalTrials.gov identifier NCT02606682). However, data from this study are not available, and further plans for the development of this compound have not been announced.

Proclara Bioscience has generated a protein that is a fusion between human immunoglobulin and a protein motif, which they report has general binding to amyloid proteins (called GAIM, short for General Amyloid Interaction Motif). When tested in an α‐synuclein overexpressing mouse, it is reported to reduce α‐synuclein aggregation and protect nigrostriatal neurons. These results have prompted the initiation of a multiple‐dose safety study of NPT088 (ClinicalTrials.gov identifier NCT03008161).

### Autophagy Enhancers

Autophagy is a collective term for different molecular pathways by which cells degrade unwanted organelles and proteins, including misfolded and aggregated α‐synuclein.^30^ After the autophagosome, a cytoplasmic organelle with a double membrane structure surrounding a lumen, has formed around its target it can merge with a lysosome to form an autophagolysosome where lysosomal enzymes degrade the cargo. When autophagy is inhibited, aggregated α‐synuclein has been shown to accumulate intracellularly and also undergo secretion via the autophagy‐secretory pathway.^31^ Not only can intracellular accumulation of aggregated α‐synuclein potentially damage or disable the host neuron but also secretion of aggregated α‐synuclein into the extracellular space has been suggested to promote cell‐to‐cell propagation of α‐synuclein pathology to neighboring cells.^32^ A key determinant of activity of the autophagic pathway is activity of the mammalian target of rapamycin, which on activation by phosphorylation will in turn inhibit autophagy.^33^ Thus, the mammalian target of rapamycin inhibitors such as rapamycin have been shown to enhance autophagy and reduce α‐synuclein pathology in model systems.^34^ However, the toxicity of rapamycin makes it a poor candidate for development as a disease‐modifying drug in PD. A recent study showed that MSDC‐0160, which is an inhibitor of the mitochondrial pyruvate carrier that was originally developed for type II diabetes, also is an effective inhibitor of the mammalian target of rapamycin by altering cellular metabolism.^35^ By inhibiting the mitochondrial pyruvate carrier, neuronal α‐synuclein toxicity is reduced in a *Caenorhabditis elegans* model. Because of a favorable safety profile of MSDC‐0160 and its documented ability to access the central nervous system in humans, it is a candidate for clinical development in PD, pending demonstration that it also reduces α‐synuclein pathology in mammalian disease models.

A third approach that might result in increased autophagy of misfolded α‐synuclein has emerged in recent years. Several inhibitors of c‐Abl (ABL1, Abelson tyrosine kinase), which is a member of the Abl family of nonreceptor tyrosine kinases, are currently approved as treatments for various forms of leukemia. This kinase has multiple targets that are involved in a wide variety of functions ranging from cell growth, shape, and migration, and in the nervous system it appears to influence normal processes such as neurogenesis and neurite extension.^36^ In laboratory models of PD, c‐Abl inhibitors have been shown to inhibit protein aggregation and neurodegeneration, with some studies suggesting that the effects are on mitochondrial function and posttranslational modifications of α‐synuclein and others indicating that autophagic pathways are enhanced.^36^, ^37^ Although the precise mechanisms of action are still debated, the preclinical evidence for efficacy was sufficiently compelling for a small safety trial of the c‐Abl inhibitor nilotinib to be performed recently. In a nonblinded safety trial, without a placebo control group, patients with dementia with Lewy bodies and PD and dementia were, in some cases, reported to exhibit some improvement in motor function within 6 months of starting treatment.^38^ Despite the significant caveats with this trial, which have been summarized elsewhere,^39^ the results have triggered further trials with nilotinib in PD, where issues regarding brain access and target engagement of the drug also will be addressed (ClinicalTrials.gov identifier NCT03205488). Although the outcomes of these nilotinib trials will be important in guiding the future of c‐Abl inhibition as a potential strategy for disease modification in PD, there are other c‐Abl inhibitors available that might exhibit better brain penetrance and greater specificity for the c‐Abl kinase that could be more suitable. Further animal studies exploring and comparing these inhibitors as well as defining the mechanisms of action are clearly warranted.

### Reducing Availability of Extracellular α‐Synuclein Aggregates

A promising approach to reducing extracellular α‐synuclein that has already entered the clinical trials stage is immunotherapy. Both active (immune system stimulation) and passive (direct antibody administration) immunotherapies are being developed. Multiple preclinical studies have established that α‐synuclein antibodies can decrease α‐synuclein aggregation in α‐synuclein overexpressing mice and prevent behavioral deficits.^40^ These antibodies presumably target extracellular α‐synuclein, as they are precluded of entering cells.^41^

The first α‐synuclein‐based therapeutic candidate for PD, PRX002 developed by Prothena, entered clinical trials in 2015. PRX002 is a humanized IgG1 monoclonal antibody directed against epitopes near the C‐terminus of α‐synuclein. It was derived from the murine monoclonal antibody 9E4, which was shown to reduce α‐synuclein accumulation and to affect behavioral deterioration in transgenic animal models of synucleinopathy.^42^ A single ascending‐dose study in healthy volunteers demonstrated good safety and tolerability profiles at doses up to 30 mg/kg, a serum half‐life of 18.2 days, and dose‐dependent lowering of “free” α‐synuclein, which was maximal at a dose of 30 mg/kg and persisted for 2 to 4 weeks after a single infusion.^43^ The acceptability profile was replicated in a multiple‐ascending dose study in PD patients,^44^ along with the demonstration of CSF antibody concentrations of about 0.3% of those achieved in plasma, consistent with the behavior of other therapeutic antibodies in development. However, no alteration in the level of α‐synuclein could be demonstrated. This latter observation was attributed to the relatively low affinity of the antibody for monomeric, as opposed to aggregated, forms of α‐synuclein, which are of very low abundance in CSF. A phase 2, multinational study of PRX002/RO7046015 in recently diagnosed PD patients was initiated in June of 2017 in collaboration with Roche (ClinicalTrials.gov identifier NCT03100149).

Another anti‐α‐synuclein monoclonal antibody currently in development is BIIB054. In contrast to PRX002/RO7046015, BIIB054 is a fully human IgG1 monoclonal antibody directed at an epitope near the N‐terminus of α‐synuclein. BIIB054 was originally isolated from B‐cell lines derived from neurologically healthy individuals in the laboratories of the Swiss biotech company Neurimmune. BIIB054 is highly selective for aggregated, presumably pathological forms of α‐synuclein, with very little binding affinity for monomeric α‐synuclein.^45^ BIIB054 binds to α‐synuclein in tissue sections and extracts from PD and DLB, but not in unaffected brains, and prevents the reduction in dopamine transporter induced by injection of preformed α‐synuclein fibrils into mouse brains. A phase 1, single ascending‐dose study of BIIB054 in healthy volunteers and PD patients recently concluded. BIIB054 was well tolerated at single doses up to 90 mg/kg, had a serum half‐life of 28 days, and achieved concentrations in healthy volunteer CSF of ∼0.2% those seen in plasma.^46^ Single doses of BIIB054 up to 45 mg/kg were well tolerated in PD patients, and the pharmacokinetic profile was similar to that seen in healthy volunteers. The CSF:plasma ratio of BIIB054 in PD patients was 0.4%.^47^ A multinational phase 2 study of BIIB054 in recently diagnosed PD patients was initiated by Biogen in November 2017 (ClinicalTrials.gov identifier NCT03318523).

There are additional anti‐α‐synuclein monoclonal antibodies in earlier stages of development. The monoclonal antibody MEDI1341, which reportedly retain reduced immune effector function, is being developed by a collaboration between AstraZeneca and Takeda. MEDI1341 has entered phase 1 testing in healthy volunteers in October 2017 (ClinicalTrials.gov identifier NCT03272165). BAN0805 is a monoclonal antibody targeted at α‐synuclein oligomers and protofibrils being developed in collaboration between Bioarctic and Abbvie. At the present time, no information is publicly available regarding the development status of this antibody.^48^

The only active immunotherapy that has advanced to clinical settings is AFFITOPE PD03A, a synthetically produced vaccine containing an α‐synuclein‐mimicking peptide to elicit an α‐synuclein antibody response. Affiris AG has recently completed a randomized, placebo‐controlled, patient‐blinded phase 1 trial assessing the safety and tolerability of repeated subcutaneous administration 15 or 75 µg AFFITOPE PD03A to patients with early PD (ClinicalTrials.gov identifier NCT02267434). Both doses were well tolerated, and no drug‐related serious adverse events or reactions occurred. The majority of adverse events were mild and local reactions. AFFITOPE PD03A reportedly exhibited a dose‐ and time‐dependent immune response against the peptide itself and cross‐reactivity against α‐synuclein as well as antibody reactivation upon booster immunization.^49^

One concern that has been raised about immunotherapy for PD, be it active or passive, is whether antibodies will have sufficient brain penetration to achieve adequate target engagement. To date, none of the immunotherapy programs has reported an effect on levels of α‐synuclein in CSF. Importantly, the reduction of Abeta plaques in Alzheimer's disease by the antibody aducanumab provides evidence that effective CNS antibody concentrations can be achieved.^19^

### Inhibiting Cellular Uptake of α‐Synuclein

Several recent studies have indicated that the presence of misfolded α‐synuclein species in the extracellular space might lead to propagation of Lewy pathology between cells when nearby neurons take up small aggregates of the misfolded α‐synuclein, allowing it to seed further aggregation in the new host neuron. This might ultimately contribute to the progression of symptoms by more neurons becoming affected by the proteinopathy and the axonal transport of aggregates between brain regions, followed by their extracellular release can conceivably lay the foundation for the appearance of new deficits (eg, cognitive decline) as additional cerebral functions are impacted. Therefore, it is clear that inhibiting uptake of aggregated α‐synuclein from the extracellular space could be an attractive approach to therapy in α‐synucleinopathies. There are currently no public programs that are approaching clinical trials in this area, but the concept is worthy of mention because some of the mechanisms contributing to the cellular uptake of α‐synuclein are being elucidated, and they might be targeted by drugs. For example, heparan sulphate proteoglycans on the surface of cells have been found to bind amyloid proteins and promote their uptake through endocytosis.^50^ Thus, molecules that disrupt heparan sulphate proteoglycans (eg, heparin or chloral hydrate) have been shown to reduce α‐synuclein uptake in cell cultures. However, so far there are no studies using animal models showing that this potential therapeutic approach is viable. Another recent study identified the lymphocyte‐activation gene 3 (LAG3) receptor on the surface of neurons as a potential protein‐binding partner to extracellular fibrils of α‐synuclein.^51^ In a series of cell culture and animal model tests, it was found that knocking down the LAG3 receptor, or applying antibodies to LAG3, inhibited the uptake of extracellular α‐synuclein fibrils and the subsequent spread of pathological aggregates.^51^ Whether LAG3 is a good therapeutic target that can be addressed clinically remains to be seen. Even if this is not the main receptor, there exists additional protein binding partners to α‐synuclein fibrils on the surface of neurons and they might constitute interesting therapeutic targets.

## Therapeutic Approaches Targeting the *GBA* Pathway

The discovery of *GBA* mutations as a common genetic risk factor for PD originated from astute clinical observations of increased occurrence of parkinsonism in Gaucher disease patients and their obligate carrier family members.^52^, ^53^ GBA is a lysosomal hydrolase that converts glucosylceramide into ceramide and glucose. *GBA* deficiency causes Gaucher disease via the accumulation of undegraded substrates. Whereas homozygous or compound heterozygous mutations of *GBA* cause Gaucher disease, heterozygous *GBA* mutations increase the risk of developing PD and other synucleinopathies.^54^, ^55^ *GBA* mutations are the most frequent genetic risk factor for PD identified to date, with 7% to 10% of PD patients carrying 1 of approximately 300 reported *GBA* mutations.^54^, ^55^

*GBA*‐associated PD is clinically and pathologically indistinguishable from idiopathic PD. Recent evidence from clinical cohort studies indicate that PD patients with *GBA* mutations present an accelerated disease course and higher risk for nonmotor symptoms.^56^, ^57^, ^58^ Dementia is a major complication in late‐stage idiopathic PD that greatly affects quality of life and survival. Notably, *GBA* mutation carriers exhibit an earlier and more rapid cognitive decline when compared with noncarriers. The increased risk for dementia in *GBA*‐related PD is consistent with earlier studies demonstrating increased frequency of *GBA* mutations in patients with PD dementia and DLB.^59^

Full *GBA* gene sequencing is required to determine the presence of *GBA* pathogenic mutations. However, it is important to note that most individuals carrying *GBA* mutations will not develop PD,^60^ which has clear connotations for genetic counseling. The implications of *GBA* genetic testing in routine clinical practice are limited by the incomplete penetrance of the mutations and the lack of impact on practical treatment decisions. This practice will necessarily evolve as we approach an era of personalized medicine for PD and related synucleinopathies.

### Challenges Facing *GBA*‐Related Therapeutic Approaches

The main challenge facing *GBA*‐related therapeutics is the limited of understanding of the precise mechanisms by which *GBA* mutations increase the risk for synucleinopathies and accelerate disease progression. Importantly, a picture is beginning to emerge. The inverse relationship between levels of *GBA* and oligomeric α‐synuclein led to the proposal of a pathogenic feedback loop.^61^ A growing body of epidemiological, clinical, and basic science studies supports this concept.^62^, ^63^ Heterozygous *GBA* mutations can initiate a pathogenic cascade by decreasing *GBA* activity, therefore promoting substrate accumulation in specific neurons.^64^, ^65^, ^66^ Alterations in glycosphingolipid homeostasis that affect membrane composition can impair vesicular transport and lysosomal/endosomal function and promote α‐synuclein aggregation, all of which might result in synaptic dysfunction and selective neuronal demise.^62^, ^67^, ^68^, ^69^ Of note, PD patients carrying nonmutated *GBA* alleles present decreased *GBA* in the brain, CSF, and blood,^70^, ^71^, ^72^ which suggests a role for *GBA*‐related mechanisms in sporadic disease.

Additional challenges for the development of *GBA*‐related therapies include the current lack of indicators of biological activity (eg, α‐synuclein aggregation) and understanding the precise level of pathway modulation required for a therapeutic effect.

### Increasing Glucocerebrosidase (GCase) Activity

The current leading hypothesis posits that *GBA*‐mediated loss of function causes an abnormal glycosphingolipid environment, leading to cellular protein mishandling (proteinopathy) and neuronal dysfunction. In animal models of disease, decreased GCase activity results in increased CNS α‐synuclein/ubiquitin/tau aggregates and associated cognitive and motor deficits.^73^ These pathological and behavioral aberrations can be ameliorated (and even reversed) by viral gene therapy‐mediated overexpression of exogenous GCase in the CNS, which would act by restoring membrane glycosphingolipid balance (Fig. 1).^73^, ^74^, ^75^ These studies provide strong support for *GBA* augmentation as a therapy for *GBA*‐associated PD and conceivably for certain forms of sporadic PD disease. As a consequence, several therapeutic strategies aimed at increasing GCase activity in the CNS through gene therapy or small molecule activators of GCase activity are under active investigation.

During the past decade, various gene therapy clinical trials for PD have been initiated and completed. Adeno‐associated virus vector platforms provide a safe, persistent expression of biologically active proteins to target structures in the human brain.^76^ In addition, preclinical studies demonstrated that *GBA* augmentation via adeno‐associated virus 1 reversed cognitive deficits in a mouse model of Gaucher‐related synucleinopathy and decreased α‐synuclein in an A53T‐SNCA mouse model.^74^ An extensive increase in GCase activity in the CNS would be required for clinical translation of the therapeutic benefit. The optimal serotype, route of delivery, and brain distribution to critical brain regions must be further investigated prior to the development of *GBA* gene therapy for *GBA*‐related PD.

The use of brain‐penetrant small molecules has also been proposed to overcome the limited distribution of gene‐therapy approaches.^62^ Small molecular chaperones, designed to cross the blood–brain barrier, that are capable of increasing lysosomal GCase activity are being investigated as a novel therapeutic approach for PD and related synucleinopathies. The mucolytic ambroxol approved by the European Medicines Agency (EMA) was reported to increase GCase activity in mice and nonhuman primates.^77^, ^78^ Ambroxol exhibits chaperone activity resulting in improved GCase lysosomal transport and reduced α‐synuclein and S129‐phosphorylated α‐synuclein protein levels in mouse models of synucleinopathy.^77^ These results have prompted the initiation of 2 clinical trials to evaluate the safety, tolerability, and efficacy of ambroxol in PD (ClinicalTrials.gov identifiers NCT02941822 and NCT02914366).

Another class of small molecules capable of increasing GCase activity is inhibitory chaperones that stabilize the enzyme by interacting with the active site. The first GCase chaperone to undergo clinical trials for Gaucher disease was isofagomine (afegostat‐tartrate, AT2101). However, in a small trial in Gaucher patients, treatment with isofagomine did not lead to significant clinical improvement, and further clinical development for this indication was discontinued.^79^

A new generation of noninhibitory GCase chaperones is being developed to overcome the limitations of active‐site inhibitors. These compounds bind to GCase some distance from the active site and induce a conformational change that increases GCase activity and/or extends its half‐life. Two of these chemical chaperones, NCGC758 and NCGC607, have undergone medicinal chemistry optimization and testing in human midbrain dopaminergic neurons from patients with Gaucher disease or PD. These noninhibitory, brain‐penetrant chaperones reduced substrate accumulation, increased lysosomal activity, enhanced GCase translocation to lysosomes, and reversed α‐synuclein accumulation.^80^, ^81^ These drugs are not as far advanced through the development process as the inhibitory chaperones; however, some small chemical chaperones hold great promise as they can modulate both GCase activity and protein levels in the brain.

### Modulating *GBA*‐Related Glycosphingolipids (Substrate Reduction Therapy)

An alternative approach to arrest the abnormal GCase/α‐synuclein feedback loop is by modulation of glycosphingolipid turnover. Reduction of glycosphingolipids by antagonists of glucosylceramide synthase can effectively treat the visceral and hematological manifestations of Gaucher disease; however, the current approved inhibitors do not achieve effective CNS concentrations.^82^ Substrate reduction therapy does not target the mutant enzyme, but instead prevents the accumulation of lipid substrates by inhibiting their biosynthesis. Inhibition of substrate accumulation equilibrates biosynthesis with the impaired catabolism as a result of *GBA* mutation or α‐synuclein inhibition. Novel glucosylceramide synthase inhibitors with good brain penetration profiles have demonstrated improved α‐synuclein processing and behavioral outcomes in mouse models of Gaucher‐related synucleinopathy and α‐synuclein overexpression.^66^ These results provide additional evidence for the role of glycosphingolipids in the pathogenic feedback loop. These results have prompted Sanofi to initiate a double‐blind, placebo‐controlled study to assess the efficacy and safety of a glucosylceramide synthase inhibitor (GZ/SAR402671, venglustat) in PD patients carrying a *GBA* mutation (ClinicalTrials.gov identifier NCT02906020).^83^

## Therapeutic Approaches Targeting the *LRRK2* Pathway

Mutations in the gene‐encoding leucine‐rich repeat kinase 2 (*LRRK2*) were initially identified in a series of families with autosomal dominant parkinsonism. Some *LRRK2* mutations are relatively common; for instance, the G2019S variant is present worldwide and has a high prevalence in North Africa where it accounts for more than 30% of PD cases.^3^ Of note, *LRRK2* mutations have an age‐dependent incomplete penetrance (ie, some individuals live until old age without any clinical signs of PD) and noncoding polymorphisms close to the *LRRK2* locus can also act as risk factors for sporadic disease. The clinical and pathological features of *LRRK2* patients strongly resemble idiopathic disease. However, a recent prospective longitudinal study reported a slower decline in motor scores among patients carrying a LRRK2 G2019S allele compared to idiopathic PD.^84^

The LRRK2 protein contains several domains that indicate a role in cellular signaling, including a kinase domain. In addition, LRRK2 interacts with many key proteins implicated in PD, suggesting that LRRK2 may be a central player in the pathways underlying disease pathogenesis.^85^ The current leading hypothesis postulates that increased kinase activity by *LRRK2* mutations are responsible for PD. However, it remains unclear whether elevated LRRK2 kinase activity in neurons (where it is expressed at low levels) or in immune cells is the key driver of PD pathogenesis.^86^ Notwithstanding this gap in knowledge, LRRK2 has been proposed as a key target for therapeutic intervention and several kinase inhibitors are at various preclinical stages (Fig. 1).^87^

### Challenges Facing LRRK2‐Related Therapeutic Approaches

There are specific challenges related to developing effective therapies targeting the *LRRK2* pathway. The development of lung morphological changes after LRRK2 inhibition in nonhuman primates raised a potential safety liability for this approach.^88^ These findings prompted a concerted effort coordinated by the Michael J Fox Foundation to evaluate the effects of three structurally different LRRK2 kinase inhibitors from Pfizer, Merck, and Genentech.^89^ The abnormal accumulation of lamellar bodies in type‐II pneumocytes was replicated at high doses for all 3 compounds, indicating a LRRK2 “on target” effect. Importantly, there was no deficit in measures of pulmonary function at the highest doses, and the morphological aberrations were reversible after a 2‐week off‐dose period. These results offer a cautionary note for pharmacological inhibition of LRRK2 in humans, particularly considering the need of chronic dosing in PD. It will be critical to understand the level of pathway inhibition required to achieve a therapeutic effect and avoid possible chronic toxic effects. An alternate strategy to reduce LRRK2 activity in the CNS, while bypassing peripheral adverse effects, is the use of antisense oligonucleotides as recently demonstrated in a preclinical report.^90^

The development of LRRK2‐related therapeutics faces additional challenges similar to the above‐mentioned approaches, such as the lack of markers of PD progression and α‐synuclein aggregation. The field is actively pursuing the search of biochemical markers of LRRK2 activation status, particularly in the CNS. Changes in phosphorylation of LRRK2 targets in peripheral blood neutrophils are also being explored as a potential biomarker of drug target engagement.^91^ These biomarkers will be necessary to confirm target engagement and, potentially, to determine patient subgroups (ie, with high LRRK2 activation status) that might receive greater benefits from these therapies.

### LRRK2 Kinase Inhibitors

Although there are several companies pursuing LRRK2 kinase inhibitors, only Denali Therapeutics has announced the initiation of clinical testing. In a healthy volunteer phase 1 study, DNL201, a small‐molecule LRRK2 inhibitor, achieved on average greater than 90% inhibition of LRRK2 kinase activity at peak and greater then 50% inhibition at trough drug levels at the highest multiple dose tested in blood as measured by LRRK2 S935 phosphorylation and phosphorylation of the substrate Rab10 in the periphery. Because of the potential safety liabilities observed in preclinical studies, the FDA placed a clinical hold to impose an exposure limit sufficient to achieve 50% inhibition of LRRK2 activity on average. The company reported that the FDA lifted the hold following review of the data from the trial and additional preclinical results. The company also reported DNL201 achieved appropriate CNS penetration by measurement of the compound in CSF. Denali has recently announced that it has commenced dosing a second LRRK2 inhibitor, DNL151, in healthy volunteers.^92^

## Key Advances in PD Clinical Sciences

### PD Progression and Subtypes: Standardized Natural History and Biomarker Studies

Understanding the clinical meaningfulness of the effects of “symptomatic” medications such as levodopa and dopamine agonists in PD is fairly straightforward because of the rapid and dramatic nature of the clinical response. However, for agents aimed at slowing PD progression, not necessarily associated with short‐term clinical improvement, detecting a clinically meaningful effect presents a significant challenge because of the slow rate and variability of the progression of symptoms between individuals.^93^ In this setting, the use of biomarkers to select and stratify patient populations, as well as to document biological effects of candidate drugs, assume critical importance in the drug development efforts. The availability of large, prospective natural history datasets that contain detailed clinical, imaging, genetic, and fluid biomarker data is key to efforts to develop disease‐modifying therapies for PD, just as the Alzheimer's Disease Neuroimaging Initiative has so well served the cause of drug development for Alzheimer's disease.^94^, ^95^ The Parkinson Progression Markers Initiative, funded by the Michael J Fox Foundation, has enrolled more than 1000 PD and control participants, with some now followed for more than 5 years.^96^ Recently, the Critical Path for Parkinson's initiative, sponsored by Parkinson's UK and the Critical Path Institute in the United States, has started to standardize datasets to create a common database for the combined analysis of Parkinson Progression Markers Initiative along with 4 ongoing U.K. natural history studies.^97^ The combined database will contain information on more than 6000 early PD patients, enabling a depth and precision of analysis and modeling and simulation activities previously not possible. A demonstration of the power and utility of the consortium approach was the issuance of letters of support by both the FDA and the EMA for the use of dopamine transporter imaging as a trial enrichment tool for eliminating subjects without apparent striatal dopamine nerve terminal loss from PD clinical trials.^98^, ^99^ In addition, disease progression models derived from the Critical Path for Parkinson's and other databases may enable a better understanding of the progression of PD and lead to the development of new comprehensive outcome measures that describe the totality of the patient experience. These precompetitive initiatives provide large, prospectively collected, standardized datasets of key relevance for the advancement of clinical trial methodology and supporting regulatory approval.

### The Concept of Prodromal PD and Its Application to Prevention Trials

Successful treatment of PD with a “neuroprotective” or “disease‐modifying” agent might slow or halt the progression of PD symptoms, but may not allow for restoration and repair of damage done to the brain and peripheral nervous. Numerous reviews have pointed out that α‐synuclein accumulation in neurons likely begins years if not decades before the emergence of motor symptoms. Once motor symptoms do emerge, even more time may pass before they are recognized as parkinsonism.^100^ However, unlike Alzheimer's disease, where similar observations have inspired the initiation of “prevention” trials in individuals either genetically at risk (DIANE) or bearing imaging evidence of brain amyloid pathology (A4), prodromal PD is characterized by an accumulation of relatively nonspecific and unrelated symptoms and signs such as hyposmia, rapid eye movement sleep behavior disorder, and autonomic dysfunction, thus making recognition of the prodromal PD difficult in the general practice environment. Furthermore, we lack a biomarker of PD pathology that has the discriminative and predictive abilities of amyloid PET imaging.^16^, ^17^ To enable the conduct of true prevention trials in PD, not only do we need strongly predictive biomarkers but also we need to educate general internal medicine, geriatric, and primary care physicians as well as the lay community about the importance of recognizing prodromal PD.

### Digital Drug Development Tools for PD Drug Development

Another difficulty for the development of effective therapies for diseases with slow and variable progression such as PD is the lack of sensitive and objective progression measures. In addition, PD is characterized by substantial inter‐ and intrapatient variability in clinical symptoms. The past few years have seen an explosion in the application of mobile and digital technologies in the clinical trials arena.^101^ The use of smartphone technology as well as digital biosensors that can generate fine‐grained, continuous outputs of activity and gait motor function both in the clinic and in the external “real world” environment presents the opportunity to (1) remove the subjectivity from in‐clinic assessments, (2) increase the density of observational data by extending the investigator's range of observation to the home environment, and (3) reduce costs and the patient/caregiver burden of clinical trials by transferring the locus of assessment out of the clinic. As promising as the application of such digital drug development tools might be, several challenges remain to their universal incorporation in clinical trials.^102^ Currently, several groups are gathering data on PD patients using digital technologies, but no harmonization efforts to address outstanding challenges are underway. Harmonization will need to be addressed to demonstrate to regulators and agencies that pay for medical care the validity and clinical meaningfulness of new digital measures.

## Coda: Will We Know It When We See It?

Rapid progress in identifying human genetic risk associated with neurodegenerative diseases has revealed numerous genes involved in PD. Mutations of these genes cause or are major risk factors for PD. Biological understanding of the function of these genes and their roles in disease pathogenesis are of utmost importance to develop therapies, and there still remains a great deal of work before we have fully understood the roles of the proteins α‐synuclein, *GBA* and *LRRK2* in PD. We have highlighted 3 of the discoveries of genetic variants in α‐synuclein, GCase, and LRRK2 that prompted attempts to develop new therapies, some of which are now entering the clinical stages. We are at an exciting juncture in the journey to developing pharmacological treatments based on knowledge of disease pathology and genetics to potentially slow the PD progression. Despite this optimistic outlook, we need to underscore that the development of these agents is still in its infancy. To date, none of these new agents has truly achieved what could be referred to as “proof of concept,” that is, accretion of sufficient data to convince industrial or government sponsors to invest in phase 3 clinical trials.^103^ Accumulating observational data; new molecular, biomarker, and clinical insights; global collaborations; and emerging technologies should enable more efficient and productive clinical trials. We can remain optimistic that the pace of change in the development of PD therapeutics will continue to accelerate and hopefully lead to the first treatment that slows PD progression within the coming decade.

## Author Roles

1) Research project: A. Conception, B. Organization, C. Execution; 2) Statistical Analysis: A. Design, B. Execution, C. Review and Critique; 3) Manuscript: A. Writing of the first draft, B. Review and Critique.

S.P.S.: 3A, 3B

J.M.C.: 3A, 3B

P.B.: 3A, 3B

## Full financial disclosure for the previous 12 months

S.P.S. is an employee and stockholder of Sanofi S.A. J.M.C. is an employee and stockholder of Biogen, Inc. P.B. has received commercial support as a consultant from Renovo Neural, Inc., Roche, Teva Inc., Lundbeck A/S, AbbVie, Neuroderm, Cellular Dynamics International, ClearView Healthcare, FCB Health, IOS Press Partners, and Capital Technologies, Inc. He has also received commercial support for grants/research from Renovo and Teva/Lundbeck. P.B. has ownership interests in Acousort AB.
